# Exploring biological interaction networks with tailored weighted quasi-bicliques

**DOI:** 10.1186/1471-2105-13-S10-S16

**Published:** 2012-06-25

**Authors:** Wen-Chieh Chang, Sudheer Vakati, Roland Krause, Oliver Eulenstein

**Affiliations:** 1 Department of Computer Science, Iowa State University, Ames, IA, 50011, USA; 2 Department of Computer Science, Free University of Berlin, 14195 Berlin, Germany; 3 Department of Computational Molecular Biology, Max Planck Institute for Molecular Genetics, 14195 Berlin, Germany; 4 Current address: Luxembourg Centre for Systems Biology, University of Luxembourg, L-4362 Esch-sur-Alzette, Luxembourg

## Abstract

**Background:**

Biological networks provide fundamental insights into the functional characterization of genes and their products, the characterization of DNA-protein interactions, the identification of regulatory mechanisms, and other biological tasks. Due to the experimental and biological complexity, their computational exploitation faces many algorithmic challenges.

**Results:**

We introduce novel weighted quasi-biclique problems to identify functional modules in biological networks when represented by bipartite graphs. In difference to previous quasi-biclique problems, we include biological interaction levels by using edge-weighted quasi-bicliques. While we prove that our problems are NP-hard, we also describe IP formulations to compute exact solutions for moderately sized networks.

**Conclusions:**

We verify the effectiveness of our IP solutions using both simulation and empirical data. The simulation shows high quasi-biclique recall rates, and the empirical data corroborate the abilities of our weighted quasi-bicliques in extracting features and recovering missing interactions from biological networks.

## Introduction

Cellular processes such as transcription, replication, metabolic catalyses, or the transport of substances are carried out by molecules that are associated in functional modules, and are often realized as physical interaction within protein complexes. These physical interactions form molecular networks. Analyzing these networks is a thriving field (e.g. [1]) and has extensive implications for a host of issues in biology, pharmacology [1], and medicine [2]. Capturing the modularities of molecular networks accurately will gain insights into cellular processes and gene function. Yet, before such modularities can be reliably inferred, challenging computational problems have to be overcome.

These computational problems typically result from incomplete and error-prone networks that largely obfuscate the reliable identification of modules [3,4]. Often, molecular interactions can not be measured to the accuracy of the genome sequences, leaving some guesswork in identifying modularities correctly. Some molecular interactions are highly transient and can only be measured indirectly, while others withstand denaturing agents. Functional interaction does not even have to be realized via physical interactions. Thus, computational methods for capturing modularity can not directly rely on presence or absence of interactions in molecular networks and need to be able to cope with substantial error rates.

**Figure 1:**
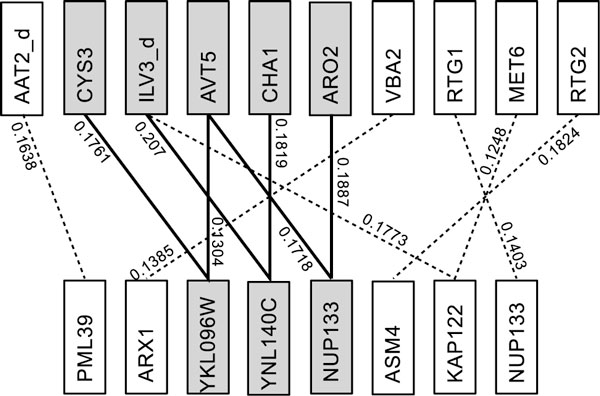
**An example of a quasi-biclique**. A quasi-biclique (darker nodes and solid edges) identified from a gene interaction network in one of our experiment sets where the edge weights are interaction scores. The bipartite graph is unweighted if only the existence of edges are considered.

Unweighted quasi-biclique approaches have been used in the past to identify modularity in protein interaction networks when presented as bipartite graphs that are spanned between different features of proteins, e.g. binding sites and domain content function [3,5]. An example is depicted in Figure 1. While these approaches aim to solve NP-hard problems using heuristics, they were able to identify some highly interactive protein complexes [6,7].

Unweighted quasi-biclique approaches are sensitive to the quantitative uncertainties intrinsic to molecular networks. Interactions are only represented by an unweighted edge in the bipartite graph if they are above some user-specified threshold. Therefore, unweighted quasi-biclique approaches are prone to disregard many of the invaluable interactions that are below the threshold, and treat all interactions above the threshold the same. Further, some interactions may or may not be represented due to some seemingly insignificant error in the measurement. Consequently, many crucial modules may be concealed and remain undetected by using unweighted quasi-biclique approaches.

Here we introduce novel weighted quasi-biclique problems by using bipartite graphs where edges are weighted by the level of the corresponding interactions, e.g., Figure 1. We show that these problems are, similar to their unweighted versions, NP-hard. However, in practice, exact Integer Programming (IP) formulations can efficiently tackle many NP-hard real-world problems [8]. Therefore, we describe exact Integer Programming (IP) solutions for our weighted quasi-biclique problems. Furthermore our IP solutions exploit the sparseness of molecular networks when represented as bipartite graphs. This allows to verify the ability of our IP solutions using a moderately sized genetic network [9], and simulation studies. In addition our IP solutions can provide exact results for instances of the unweighted quasi-biclique approaches that were previously not available.

### Related work

Maximal bicliques in biological networks are self-contained elements characterizing functional modules. In protein interaction networks they manifest as interactive protein complexes (e.g., [3,7]). *Bipartite graphs *are graphs whose vertices can be bi-partitioned into sets *X *and *Y *such that each edge is incident to vertices in *X *and *Y*. A biclique is a subgraph of a bipartite graph where every vertex in one partition is connected to every vertex in the other partition by an edge. A biclique is *maximal *if it is not properly contained in any other biclique, and it is *maximum *if no other maximal bicliques have larger total edge weights. The problem of finding maximum bicliques is well studied in the literature of graph theory and is known to be NP-complete [10] and effective heuristics for this problem have been described and used in various applications [11]. However, bicliques are too stringent for identifying modules in real world networks [12]. For example a module is not identified through a biclique that is incomplete by one single edge. *Quasi*-*bicliques *are partially incomplete bicliques that overcome this limitation. They allow a specified maximum number of edges to be missed in order to form a biclique [13]. While quasi-bicliques are less stringent for the identification of modules, they might contain genes that are interacting with only a few or none other of the genes. Such situations occur when the missing edges are not homogeneously distributed throughout. The *δ*-*quasi*-*bicliques *(*δ*-*QB*) [14] allow to control the distribution of missing edges by setting lower bounds, parametrized by *δ*, on the minimum number of incident edges to vertices in each of the vertex sets in a *δ*-QB.

### Our contributions

Here we define a "weighted" version of *δ*-QB, called *α*,*β*-weighted quasi-bicliques (*α*,*β-*WQB), to improve on the identification of modules in molecular networks by using the interaction levels between genes. Thus, *α*,*β*-WQB's may be better applicable to handle noisy data sets as they distribute the overall missing information across the vertices of the quasi-biclique as shown in our simulations. We define two versions of *α*,*β*-WQB's, each in terms of the amount of weight the quasi-biclique is allowed to miss. The two different versions of weighted quasi-bicliques provide flexibility in choosing the missing weight. For the first version, called the percentage version, we define the missing weight in terms of the percentage of number of vertices in the quasi biclique. While for the second version, called the constant version, the missing weight is defined as a constant. The need for constant version weighted quasi-bicliques arises from the fact that, for certain applications, the weight allowed to be missed does not depend on any of the graph parameters and is a constant. Finding a maximum *α*,*β*-WQB in a given edge weighted bipartite graph is NP-hard, since it is a generalization of the NP-hard problem to find *δ*-QBs in unweighted bipartite graphs [3]. We also introduce a "query" version of the maximum *α*,*β*-WQB problem that allows biologists to focus their analyzes on genes of their particular interest. Given a network and specific genes from this network, called *query*, the query problem is to find a maximum weighted *α*,*β*-WQB that includes the query. We prove this problem to be NP-hard. While the maximum *α*,*β*-WQB problem and its query version are NP-hard, we provide exact IP solutions to solve both problems. By reducing the number of required variables and exploiting the sparseness of bipartite graphs representing molecular networks, our solutions solve moderate-sized instances. This allows us to verify the applicability of *α*,*β*-WQB by analyzing the most complete data set of genetic interactions available for the Eukaryotic model organism *Saccharomyces cerevisiae*. Our results not only extract meaningful yet unexpected quasi-bicliques under functional classes, but also suggest higher possibilities of recovering missing interactions not presented in the input. A preliminary version of this work appeared in ISBRA 2011 [15]. In this paper, we extend the usage of the parameters *α *and *β *such that the edge weight threshold can be either a ratio of the *α*,*β*-WQB size or a constant. The time complexity and the application of this extended *α*,*β*-WQB are both discussed.

## Results and discussion

Before analyzing our findings in biological networks, we first introduce formal definitions of weighted-quasi bicliques (WQB) and then discuss the results of applying the WQB as a data mining tool.

### Preliminaries

A *bipartite graph*, denoted by (*U *+ *V*, *E*), is a graph whose vertex set can be partitioned into the sets *U *and *V *such that its edge set *E *consists only of edges {*u*, *v*} where *u *∈ *U *and *v *∈ *V *(*U *and *V *are independent sets). Let *G *:= (*U *+ *V*, *E*) be a bipartite graph. The graph *G *is called *complete *if for any two vertices *u *∈ *U *and *v *∈ *V *there is an edge {*u*, *v*} ∈ *E*. A *biclique *in *G *is a pair (*U'*, *V'*) that induces a complete bipartite subgraph in *G*, where *U' *⊆ *U *and *V' *⊆ *V*. Since any subgraph induced by a biclique is a complete bipartite graph, we use the two terms interchangeably. A pair (*U*, *V*) *includes *another pair (*U'*, *V'*) if *U' *⊆ *U *and *V' *⊆ *V*. In such case, we also say that the pair (*U'*, *V'*) is *included *in (*U*, *V*). A pair (*U*, *V *) is non-empty if both *U *and *V *are non-empty. A *weighted bipartite graph*, denoted by (*U *+ *V*, *E*, *ω*), is a complete bipartite graph (*U *+ *V*, *E*) with a weight function *ω *: *E *→ [0, 1].

#### Maximum weighted quasi-biclique (α,β-WQB) problem

**Definition 1 **(*α*,*β*-WQB*_P _*)). Let *G *:= (*U *+ *V*, *E*, *ω*) and *α*,*β *∈ [0, 1]. A *percentage version α*,*β*-weighted quasi-biclique, *denoted as α*,*β*-WQB*_P_*, *in G is a non-empty pair *(*U'*, *V'*) *that is included in *(*U*, *V *) *and satisfies the two properties:*

*(1) *∀*u *∈ *U' *: ∑_*v*∈*V' *_*ω*(*u*, *v*) ≥ *α *|*V'*|*, and (2) *∀*v *∈ *V' *: ∑_*u*∈*U' *_*ω*(*u*, *v*) ≥ *β*|*U'*|.

**Definition 2 **(*α*,*β*-WQB_C _). *Let G *:= (*U *+ *V*, *E*, *ω*) *and α*,*β *∈ [0, ∞). *A constant version α*,*β*-weighted quasi-biclique, *denoted as α*,*β*-WQB*_C_*, *in G is a non-empty pair *(*U'*, *V'*) *that is included in *(*U*, *V *) *and satisfies the two properties:*

*(1) *∀*u *∈ *U' *: ∑_*v*∈*V' *_*ω*(*u*, *v*) ≥ |*V'*| - *α, and (2) *∀*v *∈ *V' *: ∑_*u*∈*U' *_*ω*(*u*, *v*) ≥ |*U'*| - *β*.

In either version, the *weight *of an *α*,*β*-WQB is defined as the sum of all its edge weights.

**Definition 3 **(Maximum *α*,*β*-WQB_*P*(*C*)_). *A α,β*-*WQB*_*P*(*C*)_, is a maximum *α,β*-*WQB*_*P*(*C*) _*of a weighted bipartite graph G *:= (*U *+ *V*, *E*, *ω*), *if its weight is at least as much as the weight of any other α*,*β*-*WQB*_*P*(*C*) _*in G for given values of α and β*

**Problem 1 **(*α*,*β*-WQB_*P*(*C*)_).

*Instance: A weighted bipartite graph G *:= (*U *+ *V*, *E*, *ω*)*, and values α*, *β *∈ [0, 1]([0, ∞)).

*Find: A maximum weighted α,β*-*WQB*_*P*(*C*) _*in G*.

Note that, we use the same notation (*α*,*β*-WQB_*P*(*C*)_) for *α*, *β*-weighted quasi-biclique and maximum weighted *α*, *β*-weighted quasi-biclique problem of either version. The context in which we use the notation will make the difference clear. Also, when we just say *α*,*β*-WQB, the context will make clear if we are referring to percentage version or constant version or both.

#### Query problem

A common requirement in the analysis of networks is to provide the environment of a certain group of genes, which translates into finding the maximum weighted *α*,*β*-WQB_*P*(*C*) _which includes a specific set of vertices. We call this the query problem and is defined as follows.

**Problem 2 **(*Query*_*P*(*C*)_).

*Instance: A weighted bipartite graph G *:= (*U *+ *V*, *E*, *ω*), *values α*, *β *∈ [0, 1] ([0, ∞)), *and a pair *(*P*, *Q*) *included in *(*U*, *V*).

*Find: The α*,*β*-*WQB*_*P*(*C*) _*which includes *(*P*, *Q*) *and has a weight greater than or equal to the weight of any α*,*β*-*WQB*_*P*(*C*) _*which includes *(*P*, *Q*).

### Experiment results

Finding appropriate values for *α *and *β *is a critical part in an application. The *α *and *β *values allow the user to custom-tailor the search based on the weight distribution and the expected findings of the particular application. Typically, quasi-bicliques of different *α *and *β *values have to be analyzed by the domain experts in order to optimize the findings. We use simulated data sets to explore the problem of finding right *α *and *β *values. We then use our IP model to explore *α*,*β*-WQB's in a real world application, a recent data set of functional groups formed in genetic interactions. The filtered data set, compared to the raw data, served to investigate the role of non-existing edges in the input bipartite graph. While mathematically equivalent in the modeling step, a non-edge in a experimentally generated network represents either a true non-interaction or a false-negative. Assuming the input consists of meaningful features, our preliminary results show that *α*,*β*-WQB's may recover missing edges with potentially higher weights better than *δ*-quasi-bicliques.

### Simulations

As part of simulation studies, we try to retrieve a known maximum weighted quasi biclique from a weighted bipartite graph using both versions of *α*,*β*-WQB's. In each simulation experiment we do the following. The pair (*U*, *V*) represents the vertices of a weighted bipartite graph *G*. We randomly choose *U' *⊆ *U *and *V' *⊆ *V *as vertices of the known quasi biclique in *G*. The sizes of both *U' *and *V' *are set the same and is picked randomly, but is limited to a specific percentage of the total vertices on each side. Random edges between the vertices of *U' *and *V' *in *G *are introduced according to a pre-determined edge density *d*. The edges between vertices of *U*\*U' *and *V\V' *of *G *are also generated randomly according to a pre-determined density *d'*. The edge weights of the known quasi-biclique (*U'*, *V'*) are determined by a Gaussian distribution with a mean *mn *and standard deviation *dev*. Weights of the edges of *G *not present in the quasi-biclique are also determined by a Gaussian distribution with a lower mean *mn' *and standard deviation *dev'*. We now retrieve maximum weighted *α*,*β*-WQB*_P _*and *α*,*β*-WQB*_C _*from *G *by using specific values *α *and *β *calculated as described below.

For retrieving *α*,*β*-WQB*_P _*, the values *α *and *β *are chosen in two different ways. As part of the simulation we evaluate the performance of both methods. The first method sets both *α *and *β *to the mean of the weights of the edges of the quasi biclique. In the second method, *α *and *β *are calculated as given below:

$$\begin{array}{c} \alpha =\min\left\{C_{u^{\prime }}|C_{u^{\prime }}=\left(\sum_{v^{\prime }\in V^{\prime }}w\left(u^{\prime },v^{\prime }\right)\right)/|V^{\prime }|\text{for all }u^{\prime }\in U^{\prime }\right\}\\ \beta =\min\left\{C_{v^{\prime }}|C_{v^{\prime }}=\left(\sum_{u^{\prime }\in U^{\prime }}w\left(u^{\prime },v^{\prime }\right)\right)/|U^{\prime }|\text{for all v'}\in V^{\prime }\right\} \end{array}$$

Similarly, for retrieving *α*,*β*-WQB*_C_*, the values *α *and *β *are calculated as given below.

$$\begin{array}{c} \alpha =|V|-\min\left\{C_{u^{\prime }}|C_{u^{\prime }}=\left(\sum_{v^{\prime }\in V^{\prime }}w\left(u^{\prime },v^{\prime }\right)\right)\text{for all }u^{\prime }\in U^{\prime }\right\}\\ \beta =|U|-\min\left\{C_{v^{\prime }}|C_{v^{\prime }}=\left(\sum_{u^{\prime }\in U^{\prime }}w\left(u^{\prime },v^{\prime }\right)\right)\text{for all v'}\in V^{\prime }\right\} \end{array}$$

The ILP models of the corresponding *α*,*β*-WQB problems are generated in Python, and solved in Gurobi 4 [16] on a PC with an Intel Core2 Quad 2.4 GHz CPU with 8 GB memory.

For the evaluation, let (*U"*, *V"*) represent the maximum weighted *α*,*β*-WQB returned by the ILP model. The percentage of the vertices of *U' *in *U" *is called the recall of *U'*. Similarly, the percentage of vertices of *V' *in *V" *is called the recall of *V'*. The recalls of *U' *and *V' *are our evaluation criteria. For a specific graph sizes experiments were run by varying the values *mn *and *mn'*. The values *dev *and *dev' *were set 0.1. The densities *d *and *d' *are set to 0.8 and 0.2. The experiments were run for graphs of size 16 *× *16, 32 *× *32 and 40 × 40. Each experiment is repeated thrice and the average number of recalled vertices is calculated. The recall of the experiments can be seen in Table 1. For each graph size the first two columns represent the recall values for percentage version *α*,*β*-WQB's and the third column represents the recall value for constant version *α*,*β-*WQB. As the difference between the means increases, so does the average recall. The second method of choosing *α *and *β *for percentage version *α*,*β*-WQB yields a consistently higher recall.

**Table 1:** Simulation results of *α*, *β*-WQB recall

*mn*	*mn'*	16 × 16						32 × 32						40 × 40					
		
		Method 1		Method 2		Constant		Method 1		Method 2		Constant		Method 1		Method 2		Constant	
		*A_U_*	*A_V_*	*A_U_*	*A_V_*	*A_U_*	*A_V_*	*A_U_*	*A_V_*	*A_U_*	*A_V_*	*A_U_*	*A_V_*	*A_U_*	*A_V_*	*A_U_*	*A_V_*	*A_U_*	*A_V_*
0.5	0.5	33	77	55	100	44	22	33	30	55	6	11	40	75	37	83	79	61	62
0.55	0.45	27	38	100	100	83	88	0	0	0	0	0	0	0	25	0	0	11	0
0.6	0.4	66	77	83	88	100	88	88	56	91	80	66	70	49	53	61	66	61	66
0.65	0.35	72	77	88	88	88	88	56	66	40	66	40	66	66	91	91	100	91	100
0.7	0.3	72	100	100	100	100	100	78	83	91	91	85	91	88	77	100	83	100	83
0.75	0.25	66	83	100	100	100	100	70	69	100	100	100	100	64	91	93	100	93	100
0.8	0.2	66	100	100	100	100	100	100	78	100	91	100	91	70	76	100	100	100	100

Recall of vertices in the simulation. For every experiment, the value in the *A*_*U*_(*A*_*V*_) column represents the average recall percentage of *U'*(*V'*). The results are different from the preliminary version due to randomness in the simulation experiments.

### Genetic interaction networks

A comprehensive set of genetic interaction and functional annotation published recently by Costanzo *et al*. [9] is amongst the best single data sources for weighted biological networks. The aim of our application is to identify the maximum weighted quasi-bicliques consisting of genes in different functional classes in the Costanzo dataset.

Pairwise comparisons of the total 18 functional classes provide 153 sets. For every distinct pair (*A*, *B*) of such classes, we build a weighted bipartite graph (*U_A _*+ *V_B_*, *E*, *ω*) where genes from functional class *A *are represented as vertices in *U_A _*and genes from functional class *B *are represented as vertices in *V_B_*.

The absolute values of the interaction score *ε*, are used as the edge weights. Values greater than 1 are rounded off to 1. Any gene present in both the functional classes *A *and *B *is represented as different vertices in the partitions *U_A _*and *V_B _*and the edge between those vertices is given a weight of 1. We build LP models of both *α*,*β*-WQB versions for the bipartite graphs to identify the maximum weighted quasi-bicliques.

#### Biological interpretation and examples

Genes with high degree and strong links dominate the results. In several instances, the quasi-bicliques are trivial in the sense that only one gene is present in *U'*, and it is linked to more than 20 genes in *V'*. Such quasi-bicliques are maximal by definition but provide limited insight. A minimum of *m *= 2 genes per subset was included as an additional constraint to the LP model. It might be sensible to implement such restrictions in the application in general.

We observed the following with the maximum weighted *α*,*β*-WQB*_P_*'s in the data sets. Given the low overall weight, the *α*,*β*-WQB*_P_*'s generated with the parameters *α *= *β *= 0.1 were the most revealing. Though we found many interesting quasi-bicliques in the 153 bipartite graphs, we only present a couple of them here. A notable latent set that was obtained identified genes involved in amino acid biosynthesis (SER2, THR4, HOM6, URE2) and was found to form a 4 *× *10 maximum weighted quasi-biclique with genes coding for proteins of the translation machinery, elongation factors in particular (ELP2, ELP3, ELP4, ELP6, STP1, YPL102C, DEG1, RPL35A, IKI3, RPP1A). These connections, to our knowledge, are not described and one might speculate that this is a way how translation is coupled to the amino-acid biosynthesis. In some cases the maximum weighted quasi-biclique is centered around the genes that are annotated in more than one functional class as they provide strong weights. These genes are involved in mitochondrial to nucleus-signaling and are examples where our approach recovers known facts. Using the query approach, it is possible to obtain quasi-bicliques around a gene set of interest quickly and extend the approach proteins of interest.

Maximum weighted *α*,*β*-WQB*_C_*'s generated from the data sets with parameters *α *= *β *= 5 reveal the following. Genetic interaction networks allow to study protein-coding genes as well as genes that might only code for RNAs. A noteworthy example was discovered in the comparison of genes involved in nuclear transport and those with an unknown bioprocess revealed proteins that are part of the nuclear pore transport (POM34, NUP60, NUP157, THP2 and POM152). They interact with a number of genes that are lined up on chromosome 15 (YML033W, YML034C (SRC1) and YML035C-A) as well as and YDR431W. Most of these genes they interact with are annotated as "dubious" in the current version of the Yeast Genome Database SGD [17]. SRC1 overlaps with another uncharacterized gene YML034C-A. It would be possible that locus codes of a long RNA are involved in nuclear transport.

#### Recovering missing edges

The published data sets have edges under different thresholds removed. To sample such missing edges, we calculate the average weight of all the edges removed in the 153 bipartite graphs (generated above), and the calculated average weight is 0.0522.

For each of the 153 maximum weighted quasi-bicliques of either version, the missing edges induced by the quasi-bicliques are then identified, and the average missing edge weight *e *of each is calculated. The average missing edge weight *e *is always greater than 0.0522. In other words, we observe that a missing edge in a maximum weighted quasi-biclique has a higher expected weight than the weight of a randomly selected missing edge. This happens when the *α *and *β *values are chosen to derive *α*,*β*-WQB's which are more dense in terms of weight.

We further compare *e *from our approach to *e *from the *δ*-quasi-bicliques (*δ*-QB) described by Liu *et al*. [3]. All quasi-bicliques (including exact *δ*-QB using our IP formulation) used to induce average missing edge weight *e *are:

(1) D05/M1: *δ*-QB with *δ *= 0.5 and minimum node size is 1, i.e., *m *= 1.

(2) D05/M2: *δ*-QB with *δ *= 0.5; *m *= 2.

(3) AB/M2: *α*,*β*-WQB*_P _*using the minimum average edge weights found from D05/M2 as *α *and *β*; *m *= 2.

(4) A*X*/M2: *α*,*β*-WQB*_P _*where *X *= *α *= *β *∈ {0.05, 0.1, 0.2, 0.3, 0.4, 0.5}; *m *= 2.

(5) C*X*/M2: *α*,*β*-WQB*_C _*where *X *= *α *= *β *∈ {1, 2, 3, 4, 5}; *m *= 2.

Comparing the averages of *e *from A005/M2 to A05/M2 (please see Table 2), we see a steady increase. Since *α *and *β *can be seen as expected edge weights of the resulting QB, the changing in *e *shows that QB's identify sub-graphs of expected edge weights. However, we do not see a similar pattern in the constant versions. Overall, in this particular experiment data set, the removed edge weights are at most 0.16, hence *e *can never approach closely to the parameter *α *no matter how lenient the parameters are.

**Table 2:** Missing edge recovery in a genetic interaction network

WQB*_P_*	d05/m1	d05/m2	ab/m2	a005/m2	a01/m2	a02/m2	a03/m2	a04/m2	a05/m2
avg(*e*)	0.0855	0.0844	0.0850	0.0806	0.0830	0.0867	0.0905	0.0934	0.1169

**WQB*_C_***	**-**	**-**	**-**	**-**	**C1/M2**	**C2/M2**	**C3/M2**	**C4/M2**	**C5/M2**

avg(*e*)	-	-	-	-	0.1008	0.0805	0.0809	0.0823	0.0825

A comparison of *e *under various QB parameters showing improvements of recovered edge weight expectation in *α*,*β*-WQB's.

## Method

### Time complexity

Here we prove the NP-hardness of the *α*,*β*-WQB_*P*(*C*) _problem by a reduction from the *maximum edge biclique *problem. Note that the *query*_*P*(*c*) _problem is a generalization of *α*,*β*-WQB_*P*(*C*) _problem and hence, is also NP-hard.

**Lemma 1**. *The α,β-WQB*_*P*(*C*) _*problem is NP-hard*.

*Proof*. Given a bipartite graph *G *:= (*U *+ *V*, *E*) and an integer *k*, the *maximum edge biclique *problem asks if *G *contains a biclique with atleast *k *edges. The *maximum edge biclique *problem is NP-complete [10]. Let *G' *:= (*U *+ *V*, *E'*, *ω *) be a weighted bipartite graph where *ω*(*u*, *v*) is set to 1 if (*u*, *v*) ∈ *E *or is set to 0 otherwise. Note that, there is a biclique with *k *edges in *G *if and only if the maximum weighted *α*,*β*-WQB*_P _*in *G' *has a weight of atleast *k *when *α *and *β *are set to 1. Similarly, there is a biclique with *k *edges in *G *if and only if the maximum weighted *α*,*β*-WQB*_C _*in *G' *has a weight of atleast *k *when *α *and *β *are set to 0. Therefore, the *α*,*β*-WQB_*P*(*C*) _problem is NP-hard.

We now prove that checking for the existence of a percentage *α*,*β*-WQB in a bipartite graph is NP-complete. Note that, checking the existence of a constant version *α*,*β*-WQB in a bipartite graph can be done in polynomial time. For rest of the section we only refer to percentage version *α*,*β*-WQB's.

**Problem 3 **(Existence).

*Instance: A weighted bipartite graph G *:= (*U *+ *V*, *E*, *ω*), *values α*, *β *∈ [0, 1].

*Find: If there exists a α*,*β-WQB_P _(U'*, *V') in G*.

To prove the hardness of existence problem we need some auxiliary definitions. A *modified weighted bipartite graph*, denoted by (*U *+ *V*, *E*, Ω), is a complete bipartite graph (*U *+ *V*, *E*) with a weight function Ω: *E *→ [0, 1] where, for any two edges *e *and *e'*, |Ω(*e*) - Ω(*e'*)| ≤ 1.

**Definition 4 **(Modified *α*,*β*-WQB (MO-WQB)).

*Let G *:= (*U *+ *V*, *E*, Ω) *be a modified weighted bipartite graph. A non-empty pair (U'*, *V'*) *included in *(*U*, *V*) *is a MO-WQB of G, if it satisfies the three properties: (1) *(*U'*, *V'*) *includes *(∅, *V*)*, (2) *∀*u *∈ *U' *: ∑_*v*∈*V'*_, *w*(*u*, *v*) ≥ 0, *and (3) *∀*v *∈ *V' *: ∑_*u*∈*U'*_, *w*(*u*, *v*) ≥ 0.

**Problem 4 **(One sided existence).

*Instance: A weighted bipartite graph G *:= (*U *+ *V*, *E*, *ω*), *values α*, *β *∈ [0, 1].

*Find: If there exists a α*,*β-WQB_P _*(*U'*, *V'*) *in G which includes the pair *(∅, *V*).

**Problem 5 **(Modified existence).

*Instance: A modified weighted bipartite graph G *:= (*U *+ *V*, *E*, Ω).

*Find: If there exists a *MO-WQB *in G*.

The series of reductions to prove the hardness of the existence problem are as follows. We first reduce the *partition *problem, which is NP-complete [18], to the *modified existence *problem. The *modified existence *problem is then reduced to the one sided existence problem. The one sided existence problem reduces to the existence problem.

**Lemma 2**. *The modified existence problem is NP-complete*.

*Proof*. The proof of *MO-WQB *∈ *NP *can be briefly described in the following.

Given a weighted bipartite graph *G*(*U *+ *V*, *E*, Ω) and a pair (*U'*, *V'*) included in (*U*, *V*), it can be verified in polynomial time if the pair (*U'*, *V'*) satisfies the all the *MO-WQB *constraints for *G*. So, the modified existence problem belongs to class NP. The reduction from partition problem is as follows.

We are left to show that *partition *≤*_p _MO-WQB*. Given a finite set *A*, and a size *s*(*a*) ∈ *Z*^+ ^associated with every element *a *of *A*, the partition problem asks if *A *can be partitioned into two sets (*A*_1_, *A*_2_) such that $\sum_{a\in A_{1}}s\left(a\right)=\sum_{a\in A_{2}}s\left(a\right)$.

a. Construction: Let *SUM *be the sum of sizes of all elements in *A*. Build a modified weighted bipartite graph *G *:= (*U *+ *V*, *E*, Ω) as follows. For every element *a *in *A *there is a corresponding vertex *u_a _*in *U*. The set *V *contains two vertices *v*_+ _and *v*_-_. For every vertex *u_a _*∈ *U*, Ω(*u_a_*, *v*_+_) = *s*(*a*)/(2 × *SUM*) and Ω(*u_a_*, *v_-_*) = -*s*(*a*)/(2 × *SUM*). Add an additional vertex *u_sum _*to set *U*. Set Ω(*u_sum_*, *v*_+_) to -1/4 and Ω(*u_sum_*, *v_-_*) to 1/4. Note that, the weights assigned to edges of *G *satisfy the constraint on Ω for a modified weighted bipartite graph.

b. ⇒: Let (*A*_1_, *A*_2_) be a partition of *A *such that the sum of the sizes of elements in *A*_1 _is equal to the sum of the sizes of elements in *A*_2_. Let *U*_1 _= {*u_a _*: *a *∈ *A*_1_}. The sum of weights of all edges from *v*_+ _to the vertices in *U*_1 _is equal to 1/4. Let *U' *= *U*_1 _∪ *u_sum_*. The sum of weights of all edges from *v*_+ _to vertices of *U' *is 0. Similarly, the sum of weights of all edges from *v*_- _to vertices of *U' *is 0. Thus, (*U'*, *V*) is a *MO-WQB *of *G*.

⇐: Let (*P*, *V*) be a *MO-WQB *of *G*. The edge from *v*_- _to *u_sum _*is the only positive weighted edge from vertex *v*_-_. So, *P *will contain vertex *u*_∑_. Since Ω(*v*_+_, *u_sum_*) is negative, set *P *will also contain vertices from *U *- *u_sum_*. The sum of the weights of edges from *v*_- _to vertices in *P *- *u_sum _*cannot be smaller than -1/4. Similarly, the sum of the weights of edges from *v*_+ _to vertices in *P *- *u_sum _*cannot be smaller than 1/4. So, the sum of all elements in *A *corresponding to the vertices in *P *- *u_sum _*should be equal to *SUM*/2. This proves that if *G *contains a *MO-WQB*, set *A *can be partitioned.

Hence, the modified existence problem is NP-complete.

**Lemma 3**. *The one sided existence problem is NP-complete*.

*Proof*. The proof of *one sided existence *∈ *NP *is omitted for brevity. Next we show *MO-WQB *≤*_p _one sided existence*. We prove this problem to be NP-complete by a reduction from the modified existence problem. The reduction is as follows.

a. Construction: Let *G *:= (*U *+ *V*, *E*, Ω) be the modified weighted bipartite graph in an instance of the modified existence problem. We build a graph *G' *:= (*U *+ *V*, *E*, *ω*) for an instance of one sided existence problem from *G*. Notice that the partition and vertices remain the same. If the weight of every edge in the *G *is non negative, set *α *= *β *= 0 and *ω*(*u*, *v*) = Ω(*u*, *v*) for every edge (*u*, *v*) ∈ *E*. Otherwise, set *α *and *β *to |*x*| and *ω*(*u*, *v*) = Ω(*u*, *v*) - *x *for every edge (*u*, *v*) ∈ *E*, where *x *is the minimum edge weight in *G*.

b. ⇒ and ⇐: Let (*U'*, *V*) be a *MO-WQB *of graph *G*. If weights of all edges in *G *are non negative, the constraints for both the problems are the same. If *G *has negative weighted edges, the constraints of both the problems will be the same when *α*,*β *and *ω *for the one sided existence problem instance are set as mentioned in the construction. It can be seen that there is a *MO-WQB *in *G *if and only if there is a *α*,*β*-WQB*_P _*in the graph *G' *which includes the pair (∅, *B*).

This proves that the one sided existence problem is NP-complete.

**Lemma 4**. Existence *problem is NP-complete*.

*Proof*. Given a set of vertices (*U'*, *V'*), a weighted bipartite graph *G *= (*U *+ *V*, *E*, *ω*) and values *α*, *β *∈ [0, 1], it can be verified in polynomial time if (*U'*, *V'*) is a *α*,*β*-WQB*_P _*in *G*. Thus, the existence problem belongs to NP. We now show that *One sided existence *≤*_p _existence*.

a. Construction: Let *G' *= (*U *+ *V*, *E'*, *ω*), *α'*, *β' *∈ [0, 1] be the parameters of the one sided existence problem. We build the weighted bipartite graph *G *= (*U_p _*+ *V*, *E*, *ω*) for the instance of existence problem as follows. First, set *G *= *G'*. For every vertex *u *∈ *U*, let *S_u _*denote the sum of the weights of all edges incident on *u*. Delete every vertex *u *∈ *U *whose *S_u _*is less than *α*|*V*|. Let (*U_p _*+ *V*) denote the remaining vertices, and *E' *represent the remaining edges in *G*. For the instance of the existence problem, set *α *= 0 and *β *= *β'*.

b. ⇒ and ⇐: Any *α*,*β*-WQB*_P _*in *G' *which includes (∅, *V*), is also *α*,*β*-WQB*_P _*in *G*. Consider a *α*,*β*-WQB*_P _*(*U'*, *V'*) in *G*. If *V' *= *V*, then (*U'*, *V'*) is a *α*,*β*-WQB*_P _*in *G' *which includes the pair (∅, *V*). If *V' *is not the same set as *V*, the pair (*U'*, *V*) is still a *α*,*β*-WQB*_P _*in *G' *and it includes the pair (∅, *V*).

### IP formulations for the *α*,*β*-WQB problem

Although greedy approaches are often used in problems of a similar structure, e.g., multi-dimensional knapsack [19], *δ*-QB [3], in our experiments, both greedy and randomized approach did not identify solutions close enough to the exact solutions. In our experiments, simple greedy and randomized solutions yielded accuracies ranging from 60% to 95% depending on various parameters without performance guarantee. Hence we consider that it is rather important here to find exact solutions in order to demonstrate the usefulness of *α*,*β*-WQB's. Here we present integer programming (IP) formulations solving the *α*,*β*-WQB problem in exact solutions.

Due to the similarity in formulating constraints between *α*,*β*-WQB*_C _*and *α*,*β*-WQB*_P_*, we start by formulating a solution to *α*,*β*-WQB*_P _*. Our initial IP requires quadratic constraints, which are then replaced by linear constraints such that it can be solved by various optimization software packages. Our final formulation is further improved by adopting the implication rule to simplify variables involved. This improved formulation requires variables and constraints linear to the number of input edges, and thus, suits better for sparse graphs. Throughout the section, unless stated otherwise, *G *:= (*U *+ *V*, *E*, *ω*) represents a weighted bipartite graph, and *G' *= (*U'*, *V'*) represents the maximum weighted *α*,*β*-WQB of *G *and *E' *represents the edges induced by *G' *in *G*.

#### Quadratic programming

For each *u *∈ *U *(*v *∈ *V*), a binary variable *x_u _*(*x_v_*) is introduced. The variable *x_u _*(*x_v_*) is 1 if and only if vertex *u *(*v*) is in *U' *(*V'*). The integer program to find the solution *G' *can be formulated as follows.

(1) $$\begin{array}{ccc} \text{Binary variables:} & x_{u},\;\text{s}\text{.t}\text{. }x_{u}=1\;\text{iff }u\in U^{\prime } & \text{for each }u\in U \end{array}$$

(2) $$\begin{array}{ccc} & x_{v},\;\text{s}\text{.t}\text{. }x_{v}=1\;\text{i v}\in V^{\prime } & \text{for each }v\in V \end{array}$$

(3) $$\begin{array}{ccc} \text{Subject to: } & \sum_{v\in V}\omega \left(u,v\right)\cdot x_{v}x_{u}\geq \alpha \sum_{v\in V}x_{v}x_{u} & \text{for all }u\in U \end{array}$$

(4) $$\begin{array}{ccc} & \sum_{u\in U}\omega \left(u,v\right)\cdot x_{u}x_{v}\geq \beta \sum_{u\in U}x_{u}x_{v} & \text{for all }u\in V \end{array}$$

(5) $$\begin{array}{cc} \text{Maximize:} & \sum_{\left(u,v\right)\in U\times V}x_{u}x_{v}\cdot \omega \left(u,v\right) \end{array}$$

The quadratic terms in the constraints are necessary because, *α *and *β *thresholds apply only to vertices in *U' *and *V'*. This formulation uses variables and constraints linear to the size of input vertices, i.e., *O*(|*U*| + |*V*|). Since solving a quadratic program usually requires a proprietary solver, we reformulate the program so that all expressions are linear.

#### Converted linear programming

A standard approach to convert a quadratic program to a linear one is introducing auxiliary variables to replace the quadratic terms. Here we introduce a binary variable *y_uv _*for every edge (*u*, *v*) in *G*, such that, *y_uv _*= 1 if and only if *x_u _*= *x_v _*= 1, i.e., the edge (*u*, *v*) is in *G'*. The linear program to find the solution *G' *is formulated as follows.

$$\begin{array}{ccc} \text{Binary variables:} & \text{Same as in (1) and (2)} & \end{array}$$

(6) $$\begin{array}{ccc} & y_{uv},\text{s}\text{.t}\text{.},y_{uv}=1\;\text{iff }x_{u}=x_{v}=1 & \text{for all (}u,v)\in E \end{array}$$

(7) $$\begin{array}{ccc} \text{Subject to:} & y_{uv}\leq (x_{u}+x_{v})/2 & \text{for all (}u,v)\in E \end{array}$$

(8) $$\begin{array}{cc} y_{uv}\geq x_{u}+x_{v}-1 & \text{for all (}u,v)\in E \end{array}$$

(9) $$\begin{array}{cc} \sum_{v\in V}\omega \left(u,v\right)\cdot y_{uv}\geq \alpha \sum_{v\in V}y_{uv} & \text{for all }u\in U \end{array}$$

(10) $$\begin{array}{cc} \sum_{u\in U}\omega \left(u,v\right)\cdot y_{uv}\geq \beta \sum_{u\in U}y_{uv} & \text{for all }v\in V \end{array}$$

(11) $$\begin{array}{ccc} \text{Maximize:} & \sum_{\left(u,v\right)\in U\times V}\omega \left(u,v\right)\cdot y_{uv} & \end{array}$$

Expressions (7) and (8) state the condition that *y_uv _*= 1 if and only of *x_u _*= *x_v _*= 1. Expression (8) ensures that, for any edge whose end points (*u*, *v*) are chosen to be in G', *y_uv _*is set to 1. Due to the use of *y_uv _*variables, this formulation requires *O*(|*U*||*V*|) variables and constraints.

#### Improved linear programming

Observe that constraint (7) becomes trivial if *y_uv _*= 0. In other words, this constraint formulates implications, e.g., for binary variables *p *and *q*, the expression *p *≤ *q *is equivalent to *p *→ *q*. Expanding on this idea, we eliminate the requirement of variables *y_uv _*in constraints (9) and (10) in the next formulation while sharing the rest of the aforementioned linear program.

(12) $$\begin{array}{ccc} \text{Subject to:} & \sum_{v\in V}\left(\omega \left(u,v\right)-\alpha \right)x_{v}\geq |V|\left(x_{u}-1\right) & \text{for all }u\in U \end{array}$$

(13) $$\begin{array}{ccc} & \sum_{u\in U}\left(\omega \left(u,v\right)-\beta \right)x_{u}\geq |U|\left(x_{v}-1\right) & \text{for all }v\in V \end{array}$$

There is a variable *x_v _*for every vertex *v *in *G*. There is a variable *y_uv _*for every edge (*u*, *v*) in *G *whose weight is not 0. The variable *y_uv _*is set to 1 if and only if both *x_u _*and *x_v _*are set to 1. For any vertex *u *∈ *U *(*v *∈ *V*), the variable *x_u _*(*x_v_*) is set to 1 if and only if vertex *u *(*v*) is in *G'*. Constraint (12) can also be explained as follows. If *x_u _*= 1, the constraint transforms to the second constraint in the *α*,*β*-WQB Definition. If *x_u _*= 0, constraint (12) becomes trivial. Constraint (13) can be explained in a similar manner.

#### *Generalized formulation for α,β-WQB_P _and α*,*β-WQB_C_*

Recall that the difference between the two problems *α*,*β*-WQB*_P _*and *α*,*β*-WQB*_C _*is in the edge weight summation which we can combine as the following properties: (1) ∀*u *∈ *U' *: ∑_*v*∈*V' *_*ω*(*u*, *v*) ≥ *α*_*P *_|*V'*| - *α_C_*, and (2) ∀*v *∈ *V' *: ∑_*u*∈*U' *_*ω*(*u*, *v*) ≥ *β*_*P *_|*U'*| - *β_C_*, where (*α_P_*, *β_P _*) and (*α_C_*, *β_C _*) are the parameters given in *α*,*β*-WQB*_P _*and *α*,*β*-WQB*_C _*respectively. Following the same reasoning in the previous paragraphs, linear constraints (12) and (13) are now updated as the following.

(14) $$\begin{array}{ccc} \text{Subject to:} & \sum_{v\in V}\left(\omega \left(u,v\right)-\alpha_{P}\right)x_{v}+\alpha_{C}\geq |V|\left(x_{u}-1\right) & \text{for all }u\in U \end{array}$$

(15) $$\begin{array}{ccc} & \sum_{u\in U}\left(\omega \left(u,v\right)-\beta_{P}\right)x_{u}+\beta_{C}\geq |U|\left(x_{v}-1\right) & \text{for all }v\in V \end{array}$$

As a results, the problem instance is a *α*,*β*-WQB*_C _*problem if (*α_P_*, *β_P _*) = (1, 1), and it is a *α*,*β*-WQB*_P _*problem if (*α_C_*, *α_C_*) = (0, 0). Note that the formulation does not require either condition to present; it essentially defines a generalization of *α*,*β*-WQB problems when all 4 parameters are valid and non-zero.

If there are *n *vertices in *U *and *m *vertices in *V*, there will be a total of *m *+ *n *+ 2*k *constraints and *m *+ *n *+ *k *variables where *k *is the number of edges whose weight is not equal to 0. The above formulations can be extended to solve the query problem by adding an additional constraint *x_v _*= 1 to the formulation, for every vertex *v *∈ *P *∪ *Q*. Similar constraints also help us explore sub optimal solutions, e.g., excluding known vertices in subsequent solutions, or provide a lower-bound of required query items in the optimal solution.

## Conclusions

We address noise and incompleteness in biological networks by introducing graph-theoretical optimization problems that identify variations of novel weighted quasi-bicliques. These quasi-biclique problems incorporate biological interaction levels in different analytical settings and exhibit improvements over un-weighted quasi-bicliques. To meet demands of biologists we also provide a query version of (weighted) quasi-biclique problems. We prove that our problems are NP-hard, and describe IP formulations that can tackle moderate sized problem instances. Simulations and empirical data solved by our IP formulation suggest that our weighted quasi-biclique problems are applicable to various other biological networks.

Future work will concentrate on the design of algorithms for solving large-scale instances of weighted quasi-biclique problems within guaranteed bounds. Greedy approaches may result in effective heuristics that can analyze ever-growing biological networks. A practical extension to the query problem is the development of an efficient enumeration of all maximal *α*,*β*-WQB's.

## Competing interests

The authors declare that they have no competing interests.

## Authors' contributions

WCC and SV were both responsible for developing the solution, carrying out experiments, and writing of the manuscript. RK performed the experimental evaluation, analysis, and contributed to the writing of the manuscript. OE supervised the project and contributed to the writing of the manuscript. All authors read and approved the final manuscript.
